# Supplementary material for: Are carbon emissions trading and green financial instruments synergistic? -Comprehensive quantitative research based on content analysis
Source: PLoS One. 2024 Mar 7;19(3):e0298601. doi: 10.1371/journal.pone.0298601 (PMC10919601; doi:10.1371/journal.pone.0298601)
Supplement: S1 Data — (ZIP) [file pone.0298601.s001.zip › Data/地级市Z_F工作报告.zip!/║┌┴·╜¡/║╫╕┌╩╨/└┤╫╘╣1⁄2╓┌║┼í╛┬φ┐╦╩2╛▌═°í┐_2014.docx]

各位代表：

　　现在，我代表市人民政府向大会作工作报告，请予审议。并请各位政协委员和其它列席人员提出意见。

　　一、2013年工作回顾

　　过去的一年，我市经济社会发展遇到了前所未有的困难。国有煤矿效益下滑，地方煤矿持续停产，主要工业品市场萎缩，翘尾因素影响尚未消除，经济下行压力加大；财政刚性支出增大，多年积累的社会矛盾集中凸显，洪灾雪灾造成的损失重大，制约发展的不利因素增多。面对严峻形势，我们在省委、省政府和市委的正确领导下,紧紧依靠全市人民，坚持用发展和改革的办法解决前进中的困难和问题，虽然经济增长低于年初预期目标，但在困境中我们仍然实现了保工资、保运转、保民生、保重点、保发展，我市经济社会发展基本面向好。预计全市地区生产总值实现320亿元，同比下降9.5%；固定资产投资184.8亿元，下降5.4%；产业项目投资占固定资产投资的比重超过70%，继续保持全省前列；公共财政收入20.49亿元，下降24.59%；社会消费品零售总额107.7亿元，增长11%；利用外资6000万美元，增长15.4%；城镇居民人均可支配收入16020元，增长1.9%；农民人均纯收入14900元，增长1.4%。体现发展质量和后劲的指标有所提升，财政收入占GDP、税收占财政收入、非公经济产业投资占产业投资和民生投入占财政支出的比重，分别达到12.6%、72.7%、77.3%和76.7%。

　　——产业项目建设加快。理清了发展思路，细化了发展措施，召开了新一轮产业项目建设动员大会，出台了推进产业项目和园区建设等8个重要文件。全年开复工项目171个，其中非煤项目和资源深加工项目均超过70%。煤化工、石墨深加工和绿色食品深加工三大主导产业链条继续延伸，战略性新兴产业开始起步。中海油化肥、征楠煤化工、万源稻米深加工、经纬木糖醇、明珠大米蛋白等重点项目取得突破性进展。中铁石墨深加工、中石化乙二醇等重大项目前期工作正在积极推进。

　　——三次产业扎实推进。农村经济稳步发展。坚持推进农业“八化”建设，切实提高了农业生产力水平，改善了农业生产关系，促进了农业农村经济发展。粮食产量在大灾之年达到25亿斤。种植业结构进一步优化，全市及绥滨县水稻种植比重分别达到56%和82%，均列全省市县第一位。推广适用新技术25项，农业科技水平进一步提升。加快推进农业产业化，市级以上龙头企业达到57户，绿色食品产业实现销售收入120亿元，农民专业合作社达到312家，土地规模经营面积达到282万亩。加强农田水利建设，完成了江萝、德龙、福兴、敖来和新华灌区等26项重点水利工程的年度任务。城乡一体化和新农村建设不断深化，星级村建设位列全省第二位。工业经济困中企稳。在市场和价格“双下降”压力下，加大扶企助企力度，实行市级领导包企责任制，出台了促进工业稳定增长的“八条”政策措施，减免小微企业税费8500万元，通过工业企业助保金平台投放贷款4亿多元，协调投放贷款60多亿元，最大限度地遏制企业亏损和效益下滑。第三产业稳中有升。着力发展商贸业，益华城市综合体开工建设，萝北凤翔商贸城建成营业，大连万达城市综合体项目积极推进。巩固发展旅游业，黄金古镇、金顶山景区等升级项目顺利完工，太平沟、清源湖被评为国家级风景区。对俄开放取得突破，名山口岸正式开办异地办照业务，俄方入境“落地签”争取成功。

　　——人民生活持续改善。130件惠民实事按计划推进，各项民生承诺全部兑现。保障性住房建设实现突破。坚持“以安置促建设，以建设保安置”，2.6万套煤矿棚改住宅楼竣工，回迁安置2.1万户，是前5年的2.5倍。3180套城市棚改住宅楼、2000套廉租房和公租房完成主体工程。农村泥草房和危房改造4600多户。社保标准不断提高。企业退休人员基本养老金由月人均1430元提高到1665元，“五七工”和“家属工”养老金从684元提高到920元；城乡低保月人均保障标准从326元提高到386元；农村“五保户”集中供养标准由4000元提高到4600元；“新农合”、城镇医保由240元提高到280元，困难群众大病二次救助上限由1万元提高到3万元。最低工资标准从700元提高到1050元；社区主任、副主任生活补贴每月增加200元。就业形势总体向好。省级创业型城市建设稳步推进，帮助350多名高校毕业生、100多名残疾人实现就业，为1007名创业人员发放小额担保贷款5035万元，为851名农村妇女创业提供小额贴息贷款3320万元。新增就业2.1万人，城镇登记失业率控制在4%以内。

　　——城市建设管理加强。深入实施五大城市发展战略，完成重点工程77项。修改了城市总体规划，完善了城市道路、供热和沉陷区等专项规划，严格规划例会制度，加大规划执行力度，严肃查处了一批未批先建、批少建多和乱搭乱建等违法行为，城市规划建设有序调控。44条主干路和街巷路改造、农村电网建设和节能改造等工程年度任务全部完成，北山公园、儿童公园升级改造和南山公园新建工程全部竣工，200个“六小工程”投入使用，“五湖一河”景区主体工程全部完成，小鹤立河水库开始向工业园区供水，多年形成的近300万平方米供热缺口问题基本解决，城市承载能力有所提升。开展了市容市貌综合整治，抓绿化、治裸土、清垃圾、除冰雪，治理裸土8万平方米，植树164万株，购置清冰雪设备189台，机械化清雪率达到62%，“脏乱差”专项整治取得明显成效，强化物业管理，“弃管楼”达标管理达到50%以上，城区东部污水处理厂和两县污水处理厂投入使用，城市垃圾处理厂、市区污水管网改造基本完成，新增城市公交车100辆，新建、改建了候车亭和报刊亭，城市环境面貌有效改善。

　　——社会事业协调发展。完善了5所学校教学设施，创建了6所标准化学校、3个素质培训基地，对学校供热设施进行了升级改造，消灭了“明火炉”，办学条件进一步改善。总投资3.2亿元的鹤岗区域医疗中心投入使用，市中医院、妇幼保健院主体工程竣工，53个村卫生所标准化建设完成，兑现了城乡独生子女父母奖励费，医疗服务能力进一步提升。市博物馆和城市规划展馆正在布展，数字化图书馆、青少年科技活动中心投入使用。整合了市、矿有线电视网，新上了电视转播车，实施了城区有线电视数字化及传输系统改造。万源粮油米糠联产项目被列入国家“863”计划，全国第一个稻米综合利用工程技术中心建成，企业院士工作站通过审批，全国第一个国家石墨产品监督检验分中心通过评审，科技工作成效明显。全民健身运动蓬勃开展，新建17个群众户外健身场所，市全民健身指导中心通过国家体育总局服务质量体系认证，并被评为我省唯一的群众体育先进集体，我市运动员在各类比赛中荣获世界级金牌2枚、国家级金牌4枚和省级金牌48枚。

　　——社会形势保持稳定。启动了安全隐患和社会矛盾“双查双治双问责”活动，开展了安全生产大检查、大整顿，严格落实安全生产“四个责任”，加快地方煤矿整合整治，加大安全生产标准化建设，有效防范重特大安全事故的发生，全市各类安全事故死亡率下降73.5%，安全生产形势稳定。坚持信访源头治理，切实解决群众反映强烈的产权证、供热、拆迁、回迁和“集资借贷”等问题，使全市信访积案化解率达到80%以上，信访工作成效明显。实施了“法眼工程”，开展了“保稳定、保平安”等20多项专项行动，人民群众安全感增强。全面完善了自然灾害、突发事件、群体性事件和安全生产事故的应急处置预案，应对突发事件的能力不断提升。

　　——抗洪救灾取得全面胜利。去年，我市黑、松两江发生特大洪水，全市20个乡镇163个村6万余人受灾。在大灾面前，我市党、政、军、警、民同舟共济，众志成城，全力投入到抗洪救灾工作之中。由于科学研判，决策果断，指挥有力，抢险及时，我市没有一人伤亡，没有出现重大疫情，并在最短时间内恢复了灾区的生产生活秩序，抗洪救灾取得全面胜利。

　　统计、审计、监察、外侨、计生、老龄、气象、地震、人防、司法行政、档案、民兵、预备役等工作取得新进展,民族宗教、妇女儿童、残疾人、红十字等事业取得新进步。

　　各位代表，过去的一年,我们遇到的困难远远超过预期，取得的成绩实属来之不易。这是省委、省政府和市委正确领导的结果,是市人大、市政协监督支持的结果,是全市上下迎难而上、砥砺前行、拼搏实干的结果。在此，我代表市人民政府，向全市人民，向各民主党派、工商联、无党派人士、人民团体和社会各界人士，向中省市直各单位，向驻军指战员、武警部队官兵和公安干警，表示衷心的感谢并致以崇高的敬意！

　　在看到成绩的同时，我们也清醒地认识到，我市在发展中还存在一些问题和不足，主要是：经济发展速度不快，质量和效益还有待于提升，潜力和优势没有充分释放出来；产业结构不尽合理，“一煤独大”还没有从根本上改变；财源型项目支撑力弱，刚性支出增大，财政收支矛盾凸显；改善民生与群众的期待仍有差距，影响社会和谐稳定的因素依然较多；少数干部精神不振、状态不佳，执行力和落实力还需进一步加强。对这些问题我们将高度重视，采取有力措施，认真加以解决。

　　二、2014年工作总体部署和主要任务

　　2014年，是应对诸多困难和挑战，抢抓改革发展重大机遇，实现稳增长、调结构、转方式的关键一年，也是全力抑制经济下滑，实现提质增速的攻坚之年，更是鹤岗站在新起点、实现新发展、开创新局面极其重要的一年。

　　今年政府工作的总体要求是:认真贯彻落实中央、省委省政府和市委的一系列决策部署，坚持以科学发展为主题，以稳中求进为基调，以改革创新为动力，紧紧围绕国家“五大规划”和我市“8528”总体发展战略，更加注重深化改革、产业升级、民生改善、城市建设、社会治理和自身建设，着力推动结构转优、方式转变、城市转型，为全面建成小康社会奠定坚实基础。

　　今年全市经济社会发展的主要预期目标是：地区生产总值增长10%，固定资产投资增长27%，公共财政收入增长15%，社会消费品零售总额增长13%，进出口总额增长10%，城镇居民人均可支配收入和农民人均纯收入力争增长10%以上，单位GDP综合能耗下降4.3%。在抓好上述指标的同时，更要着力提升“五个比重”。

　　全面做好政府工作，必须坚持和把握好以下原则：

　　——必须坚持把党对经济工作的领导作为加快发展的根本保证。要把思想、步调和行动，统一到市委对当前形势的分析和判断上来，统一到对今后工作的部署和要求上来，一心一意谋发展,心无旁骛干事业,努力把鹤岗建设好、发展好。

　　——必须坚持把深化改革作为加快发展的强大动力。要处理好政府和市场的关系,使市场在资源配置中起决定性作用和更好发挥政府作用，加大重点领域和关键环节改革力度，坚决破除体制机制弊端，激发全社会的发展动力和创造活力。

　　——必须坚持把加快发展作为解决鹤岗一切矛盾、困难和问题的根本出路。要树立强烈的发展意识，增强加快发展的紧迫感，挖掘潜在优势，转化现实优势，创造竞争优势，好字优先，好中求快，扎实推动鹤岗经济持续健康发展。

　　——必须坚持把产业项目建设作为加快发展的重要引擎。要牢固树立“抓发展必须抓经济，抓经济必须抓产业，抓产业必须抓项目”的理念，立足于鹤岗的比较优势和客观实际，着力构建现代产业体系，引领和支撑经济转型、社会发展。

　　——必须坚持把保障和改善民生作为政府工作的出发点和落脚点。要切实解决人民群众最关心、最直接、最现实的问题，实现好、维护好、发展好最广大人民的根本利益，做到发展为了人民、发展依靠人民、发展成果人民共享。

　　——必须坚持把实事求是，一切从实际出发作为政府工作的基本要求。要在抓改革、抓发展、抓民生上，正确把握好尽力而为、量力而行的关系,既要尽最大努力，做到尽力而为,不留遗憾，又要不超越可能，做到量力而行，不留矛盾和问题。

　　——必须坚持把打基础、利长远作为施政准则。要以“功成不必在我”的胸襟，多做承上启下、继往开来的大事，多做攒后劲、见长效的实事，多做群众看得见、摸得着的好事，让我们的工作经得起实践、人民和历史的检验。

　　依据上述总体要求和原则，今年要重点做好六项工作。

　　（一）更加注重产业升级，推动经济快速发展

　　加快推进产业升级，既是转方式、调结构的内在要求，也是提升鹤岗综合经济实力的重要支撑。在工作摆布上，要把推进产业项目建设作为主攻方向，进一步做强做大工业、做强做精农业、做强做活第三产业。

　　全力推进产业项目建设。继续开展“三引进”和“四比竞赛”活动，举全市之力，坚决打好、打胜产业项目建设攻坚战。着力构建现代产业体系。坚持“抓项目向抓产业转移、抓产业向抓产业链转移、抓产业链向抓产业体系转移”的思路，围绕煤化工、石墨深加工和优质绿色食品三大主导产业、四大支柱产业和三大新兴产业，拓展延伸上下游产业链，加快形成现代产业体系，把新的增量做大做强。今年，重点实施50个总投资亿元以上项目。要大力推进煤化工产业，加快实施构建煤焦化及副产品加工利用、煤制化肥、煤制甲醇乙二醇、煤制气等4条产业链，重点抓好中海油华鹤“3052”化肥，征楠煤化工二期，鹤翔新能源，中石化、恒逸集团煤制乙二醇，南山煤制天然气等重大项目；要快速提升石墨深加工产业，加快实施构建石墨电池负极材料、密封材料、阻燃材料、锂离子蓄电池、胶体石墨等5条产业链，重点抓好中铁石墨等重大项目；要做强绿色食品产业，加快实施构建稻米深加工、玉米深加工、大豆深加工、畜牧养殖加工和林产品加工等产品产业链，重点抓好万源粮油米糠油扩建、秀泉科技、经纬糖醇、明珠生物科技、崧阳粮油等重大项目。加快产业项目库建设，要眼睛向内，依托我市现实资源、潜在优势生成项目；要眼睛向外，利用国内外两个市场、两种资源，盯住世界、国内500强企业、发达地区、新兴产业和大专院校、科研院所编制项目。市县（区）两级优质项目储备要保持500个以上。着力增强园区承载功能。以建设省级、国家级经济技术开发区为目标，市级园区重点做大做强煤化工、石墨精深加工、绿色食品和高新技术4个产业园，集中摆放事关城市转型、结构调整的主导产业和重大项目；要以建设专业化、特色化产业园为主导，县区和板块园区重点发展专业化、集群化的产业项目，打造符合鹤岗特色、产业集聚发展的平台。要扩大园区面积，强化园区供排水、电力、道路等基础设施建设，重点在项目要素优化聚集上下功夫，增强承载功能。着力提高产业招商实效。进一步创新招商方式，强化招商措施，提升招商效果。突出领导带头招商，落实落靠市级、县区领导亿元以上产业项目招商任务。认真执行《重大产业项目招商奖励办法》，营造全民招商氛围。进一步明确招商方向，盯住有实力的大企业，引进一批产业链长、对地方经济辐射带动作用大的好项目，做高产业层次，做优产业结构；抓住高技术、成长型的好项目，选择科技含量和附加值高、有市场潜力的产业项目；瞄准整体产业转移比重高的发达地区，主动承接产业转移，加速促进产业集聚和实现规模经济。

　　做强做大工业。牢固树立工业的主导地位和企业的主体地位，壮大新型主导产业，做强支柱骨干企业，全力遏制工业经济下滑局面。2014年，我市地方规模以上企业利税增长要达到20%以上，新增规模以上企业30户；非公企业增加值和税收要分别提高20%以上。要密切关注企业运行。强化对生产要素的调度指挥保障，严格落实市级领导包产业责任制，密切关注企业运行状况，全面掌握并及时解决企业面临的实际困难和经营难题。要扶持壮大骨干企业。高度关注已经建成的项目，把现有存量做大盘活。对龙煤鹤岗公司、华能鹤电等国有大企业以及非公有制骨干企业，要加大服务支持力度，尽全力和企业共克时艰。对近年竣工投产企业，积极帮助解决周转资金、原料供应、市场开发等方面的问题，尽快形成生产能力，迅速壮大骨干企业群体。对现有骨干企业，鼓励技术创新，加快技术改造，推进产业升级换代，加速裂变壮大。要深挖企业内部潜力，在支持企业生成项目上下功夫，依托现有骨干企业发展下游配套项目。要落实各项支持政策。继续落实好省、市政府新17条和新8条政策措施，采取加大金融支持、融通民间资本、加强资本运作等方式，进一步拓宽企业融资渠道。今年，重点支持2—3户企业在“新三板”上市。要加大政府财政支持力度，综合运用行政、经济等手段，支持企业发展。今年，投放企业助保金贷款力争10亿元以上。

　　做强做精农业。坚持把农业增长作为经济增长的重要拉动力量，大力发展现代农业。要促进粮食增产。优化种植结构，继续扩大水田规模，稳定玉米面积，粮食产量力争实现28亿斤。强化农业基础设施建设，重点实施14项水利工程，新增高效节水灌溉农田10万亩，改善灌溉农田11万亩。建设高标准基本农田，计划完成农村土地整治面积9万亩。推动现代农机合作社发展，提高农机装备水平，培养省级农机专业合作社6个，建设千亩农机科技示范园区21个，主要粮食作物实现全程机械化。促进农村经营体制创新，全市千亩以上种粮大户、家庭农场分别达到30个，新建农民专业合作社30个。继续推进农业科技进步，全市粮食作物模式化栽培率达到90%以上，建设农业科技园区50个，农业科技进步贡献率达到55%。加强气象服务，搞好农业防灾减灾。加强粮食仓储设施建设。要促进农民增收。贯彻落实强农惠农富农政策，重视增加农民在种植、养殖环节收入，更要引导和扶持农民从错季销售、精深加工、终端市场等各个环节增加收入。在城郊具备条件的区域，加快调整种植结构、转变生产经营方式，发展果菜、花卉、苗木、食用菌等高附加值农业。大力发展畜牧业，推进规模化、标准化养殖，做好重大动物疫病防控工作，保障畜产品质量安全，畜牧业产值增长要达到7%以上。积极发展劳务产业，维护进城务工农民合法权益，增加农民的工资性收入。要促进农业增效。重点是加快发展绿色产业和特色产业。新增省级龙头企业2户，培育市级龙头企业10户，推进农业产业化龙头企业规模化发展，新增国家级绿色食品水稻原料基地20万亩。实施网箱、围栏养殖面积4万平方米、名特优水产养殖面积4万亩、休闲游钓面积1.2万亩，创建水产健康养殖示范场5个；加快林果、北药种植、山野菜等特色产业发展。要促进农村发展。重点是加快推进城乡发展一体化和新农村建设。继续坚持“93668”发展思路，全面加快城乡统筹发展。抓住两大平原现代农业综合配套改革试验区的机遇，聚合新型城镇化、城乡发展一体化和新农村建设政策、资金和项目，以推动产城融合、提升公共服务水平、促进农业人口城镇化等为重点，结合美丽乡村、星级新农村建设，加快推进示范镇村建设。

　　做强做活第三产业。以发展现代服务业、旅游业、对外贸易为重点，提高第三产业对整体经济的贡献率。要培育现代服务业。加快推进万达、益华等城市综合体项目建设,大力引进新型商业业态,积极发展大型连锁超市,完善和提升消费市场,促进消费增长。发挥优势，搞好现代物流发展规划，积极引进大型物流项目，加快开发物流业市场，力争打造黑龙江省东部物流产业基地。大力发展电子商务、服务外包等现代服务业,着力引进咨询、评估、工程设计等生产型服务业。要发展金融业。大力实施“引金入鹤”，优化金融生态环境，深入推进银政、银企合作，支持金融机构发展壮大,努力发挥金融对经济发展的推动作用。今年，力争鹤岗市农商银行挂牌成立，结束鹤岗没有自己地方银行的历史。要壮大旅游业。结合优良的生态环境、深厚的文化底蕴和大河流、大森林、大冰雪、大矿山等资源，加大推进旅游精品工程建设和提档升级，做好凌霄峰滑雪场、摩天岭猎苑等精品项目的规划设计建设工作。依托名山旅游名镇、太平沟、金顶山、鹤立湖等核心景区，开发鹤岗特色旅游地产项目和特色旅游产品。加强旅游宣传推介力度，做好与周边城市以及俄方旅游的深度合作，共同打造旅游精品线路。要扩大对外贸易。抓住我省和蒙东地区沿边开发开放上升为国家战略的历史机遇，按照出口抓加工，进口抓落地的思路，加快萝北东辉等外经贸主体企业培育，加大地产品出口规模，地产品出口要达到3000万美元以上。推进对俄投资项目，扩大对俄经济技术合作，重点抓好鹤矿集团赴俄锰矿开发和赴俄农业种植，对俄投资项目进口要达到1200万美元以上。继续发展旅游贸易出口，贸易额达到5000万美元以上。扩大与欧美、东南亚等国家和地区的贸易往来，积极扶持企业开发中东、拉美、东盟和非洲等新兴国际市场。

　　（二）更加注重改善民生，不断增进人民福祉

　　越是在经济困难的时候,越要高度重视民生改善，越要解决好人民群众最迫切、最期盼、最忧心的事情。今年，我市投入改善民生的财力要增加10%以上。

　　统筹推进更高质量就业创业。加大就业创业政策落实力度，实施“三个千人”计划，安置1000个困难人员就业、帮助1000名高校毕业生实现就业，扶持1000人创业。加大公共就业服务力度，抓好孵化基地、见习基地、创业园区和省级充分就业社区建设，做好就业对接，就业创业培训8000人，开展好“春风行动”等公共就业服务专项援助活动，全年新增就业2.2万人，城镇登记失业率控制在4%以内。

　　统筹推进城乡社保体系建设。实施养老、医疗、失业、工伤、生育五险扩面，做好医疗保险参保和事业单位、社会团体等组织参加工伤保险，让更多的老百姓都能享受社保的好处。进一步提高社会保障水平，将失业保险待遇由每月620元提高到720元。城镇职工医疗费年度最高支付额由15万元提高到20万元，城镇居民医疗费年度最高支付额由6万元提高到8万元，城镇职工大病统筹报销比例由80%提高到87%，城镇居民门诊大病统筹报销比例由65%提高到75%，城镇基本医疗保险和工伤保险实现与省“金保工程”系统联网，实现本市住院患者医疗费用全部“即时结算”。继续提高低保标准，城乡低保人均标准从每月386元提高到430元。全面落实行业最低工资标准，适时提高低收入职工工资底线。进一步做好社会救助工作，积极筹措资金，做好各类困难群众救助工作，重点抓好四大传统节日的社会救助工作。做好灾区生活救助，确保灾民生活安定有序。加强残疾人社会保障体系和服务体系建设，推进残疾人扶贫解困工作。进一步发展敬老事业，广泛开展居家养老，构建全市社区养老呼叫平台，为居家养老提供全方位服务，居家养老城市社区覆盖面达到60%；改造郊区敬老院，增加集中供养床位，使农村“三无”老人集中供养率达60%以上。进一步加强社区建设，社区达标率要达到70%。

　　统筹推进城乡人居环境改善。要以筹措资金、落实政策、有序拆迁、加快建设、管住质量、保证安置为重点，推进保障性住房建设。煤矿棚改工程结转续建的63.7万平方米、102栋住宅楼全部竣工，公建及配套基础设施等全部完工。城市棚改拆迁2238户，回迁率100%。建设30万平方米5000套公租房，5万平方米1000套廉租房。加快推进灾后房屋重建，结合农村危房改造，完成新建房屋1771户，修缮房屋638户。

　　统筹推进教育事业公平发展。优化配置教育资源，提高教育质量，努力实现教育公平。完善5所新建公办幼儿园教学设施，建成1所早教中心。完善特殊教育学校办学条件和设施，提高残疾少儿入学率。创建2所标准化学校，完善5所学校基础设施，建设3个学校风雨操场，建成1个国家级学生劳动技术教育综合实践基地，10个市级素质教育综合实践基地。改造师专教学楼、实验楼，建设教师教育综合素质训练中心、学前教育和煤矿开采技术实训中心。提高教师素质，加大紧缺学科教师考录、招聘工作力度。进一步做好外来务工、贫困矿工、农民工子女入学和贫困学生资助工作。

　　统筹推进城乡医疗卫生事业。加强基层医疗卫生机构基础建设，改善医疗条件，提高服务水平，着力解决群众看病难、看病贵问题。深化医药卫生体制改革，巩固“新农合”制度，人均筹资达到390元，转变社区卫生服务模式，实施社区家庭医生签约服务。加快卫生基础建设，推进市妇幼保健院综合楼配套设施建设，市精神病院新址投入使用，启用市中医院大陆北小区医院综合楼，启动新建农村医学综合楼，建成医疗卫生用品洗涤中心。加强疾病预防控制工作，防止重大传染病暴发流行。加大卫生执法监督力度，严厉打击非法行医。坚持计划生育基本国策，兑现农村及城镇无业人员独生子女父母奖励费。

　　统筹推进文化体育事业发展。推动政府部门由办文化向管文化转变，尝试政府购买服务的办法，激活文艺创作、文艺演出和文艺人才队伍建设。充分发挥博物馆征集、保护、陈列和研究功能，深入挖掘鹤岗文化内涵，宣传、展示鹤岗。继续组织开展“欢乐鹤岗行”文化下基层活动，办好鹤岗市第二届少数民族文艺汇演。加快有线电视数字化进程，实现广播节目有线电视网内播出。深入开展档案馆功能建设，提高档案安全保障能力和公共服务能力。加强群众身边体育设施建设，广泛开展全民健身活动，提高竞技体育水平，建设10个农民体育健身工程和1个市区健身苑工程建设，定期免费开放体育场馆，进一步推进国民体质监测与评价常态化。

　　统筹推进科技兴市战略升级。加快科技创新体系建设，大力实施各类科技项目，培育专利优势企业、国家级高新技术企业和省级科技型中小企业，开发高新技术产品，实现高新技术产业产值增长20%以上。深化以企业为主体的产学研用结合，加强与高校院所科技合作，引进、转化、推广一批科技创新性强、技术关联度高、产业带动性大、影响辐射面广的技术成果。完善科技创新创业资源共享平台，加快高新技术企业和科技型企业研发机构建设，选择优势产业组建产业技术创新战略联盟。继续实施人才“百千万工程”和“千名专业人才深造工程”，鼓励高层次人才向一线聚集，在基层创新创业。

　　（三）更加注重城市建设，切实提升承载能力

　　加强城市建设，是塑造城市形象、提升城市品位、保障经济社会健康持续发展的前提和基础。必须用科学的理念、市场的机制，高标准高水平地规划建设城市、经营管理城市。

　　高起点规划城市。牢固树立“规划即法”理念，充分发挥规划的龙头作用，高质量完成城市总体规划修改工作，配套编制控制性详细规划、区域性专项规划和专业性专项规划。围绕推进城市转型，编制采煤沉陷区综合治理规划；围绕优化产业布局，编制重大项目和重点产业规划；围绕生态文明建设，编制生态文明建设规划；围绕畅通对外、对内通道，编制鹤岗交通运输总体发展规划；围绕城市基础设施建设，编制水、电、气、热网建设规划。要加大城市规划执行力度，坚持一张图规划，一支笔审批，坚决不允许再发生私批乱建、未批先建、批少建多等现象，切实维护城市规划的统一性和权威性。

　　高标准建设城市。加快路网、电网、水网、热网和气网等重大基础设施项目建设，完善城市功能，确保人民群众基本生活和城市扩张需求。实施路网建设。一方面，要规划建设对外交通体系，铁路、公路、水路、空路“四路同建”。铁路要积极推进鹤佳铁路扩能改造，鹤北至名山、鹤岗至南岔的铁路建设项目。公路要积极推进绥名公路改造和鹤大公路护坡改造项目，加快规划鹤伊公路提档升级。水路要积极推进萝北名山港码头改扩建工程，打通鹤岗入海口，实现江海联运。空路要积极推进鹤岗机场建设项目，近期要纳入国家通勤机场规划。另一方面，要加快城区道路升级改造和城市外环建设，实施推进“八横九纵三环”城市交通网络建设，重点建设昌南路、东山路等5条主次干道，打通城市道路瓶颈，让城市交通更顺畅；新建50条街巷道路，维修40条破损道路，建设南北红旗路2座过街天桥，让市民出行更便捷。实施电网建设。启动建设鹤岗500千伏输变电工程，完成10千伏电网和110千伏输变电等城农网改造升级工程，推进220千伏环山变扩建工程。实施水网建设。升级改造市政净水系统，进一步提升水质。新建农村饮水安全工程28处，解决1.2万人饮水安全问题。推进关门嘴子水库建设前期工作，年内完成《项目建议书》批复。加快我市与中国水务集团战略合作的步伐，全面提升我市供排水能力。实施热网建设。改造供热管网5公里，实施城市二热源改扩建工程，更新改造向阳地区老旧供热外网，推进2×30万千瓦城市热电联产项目。实施气网建设。铺设城市燃气管道35公里，新增用户5000户以上。

　　高效能管理城市。坚持建管并重的原则，进一步推动城市管理提档升级。要进一步理顺大城管体制，整合城管执法、规划、土地、建设、公安、环卫、交通等部门资源，履行“七加一”职能，对城市进行全方位、高效率的动态管理。要加大市容市貌整治力度，集中整治尘土、污水、垃圾、烟尘等环境质量问题，突出治理超载毁路、乱停乱靠、占道经营、管线散乱、违章建筑等管理无序问题，有效解决交通拥堵、道路破损、设施老化、牌匾广告不规范等城市形象问题。要加大建筑市场、房地产市场整治力度，依法坚决打击建筑质量、房屋征收拆迁、房屋销售以及建筑、开发企业拖欠农民工工资等方面的违法违规行为，将违法违规建筑商、开发商列入“黑名单”，让他们为违法违规行为付出代价。要加大对现有公园、广场的维护改造管理的力度，维修完善五指山、儿童、松鹤、北山等公园及新世纪、人民、站前、文化等广场设施，增设30座公交候车亭，亮化60条街巷道路，进一步满足市民休闲、健身、娱乐需求。要推进生态文明城市建设。继续实施工程、结构、管理三大减排措施，完成减排任务目标。要巩固省级生态县、国家级生态乡镇创建成果，加快大小兴安岭生态功能区建设，促进自然保护区提档升级，完成十里河晋升国家级湿地公园和细鳞河自然保护区建设工作，让城乡生态更优良；要抓好城乡结合部、城区废弃地、主次干道、居民区绿化，实施10万平方米裸土治理，提升城市园林绿化标准和水平，多渠道拓展城市绿化空间，让城市景观更靓丽；继续推进市区污水管网工程和城市生活垃圾无害化处理工程，实施烟尘、机动车尾气等城市环境综合治理，确保空气质量达标，让城市空气更清新。

　　高水平经营城市。坚持政府主导、市场化运作，通过经营城市土地、基础设施项目和城市存量资产，打破资金瓶颈，变财政投资为多渠道融资，变政府包建为多方共建，增强“造血”功能，实现以城养城、滚动发展。要搞好城市土地资产经营，政府要通过完善土地储备制度，切实垄断土地一级市场，经营性建设用地全部实行公开招标拍卖，土地转让全部实行挂牌交易，坚决防止土地收益流失。对已取得土地使用权满两年未动工建设的，要依法收回土地使用权，保证存量土地升值增值。要搞活城市基础设施建设经营，按照谁投资、谁经营、谁管理、谁受益的原则，采取BT、BOT等国际通行做法运作城建大项目，允许社会资本通过特许经营等方式参与城市基础设施投资和运营。要盘活城市资产存量，将基础设施经营权、冠名权、广告权实行统一管理，通过转让、拍卖、租赁等形式，对供水、排水、供气、供热、公共客运交通、污水处理和垃圾处理等基础设施存量资产进行市场化运作，提高经营城市效益。

　　（四）更加注重社会治理，营造安全稳定环境

　　社会治理事关社会安全稳定，事关人民安居乐业。要从改革社会治理方式、提升社会治理能力的高度出发，最大限度地维护安定和谐的社会局面，不断提高社会治理科学化水平。

　　切实抓好安全生产工作。筑牢“红线”意识，坚持“科学发展、安全发展”，深入开展全市安全隐患排查、问题治理、责任追究专项行动，严防各类安全事故发生。严格责任落实。强化安全生产“四个责任”，按照“党政同责”、“管生产必须管安全、管业务必须管安全、管行业必须管安全”及“一票否决”原则，加强执法监察，坚决遏制重特大事故发生。严守安全法规。进一步强化安全设施和职业卫生“三同时”制度落实，加大安全生产宣传教育培训力度，加快推进安全生产标准化建设。认真执行“双七条”规定，健全完善地方煤矿安全避险“六大系统”，加快推进煤矿集团化和质量标准化建设，完成年度煤矿关闭和资源整合任务。要以单井安全达标为根本标准，不允许简单拼凑产能整合。严治安全隐患。按照“全覆盖、零容忍、严执法、重实效”的原则，全面排查治理煤矿、非煤矿山、危险化学品、烟花爆竹、建筑施工、道路（水上）交通、旅游、特种设备、人员密集场所、城市燃气等领域存在的安全问题。继续加强校车、校园安全管理。不断完善安全生产应急管理机构、救援队伍、应急预案及实战演练，全面提高应急能力。加强森防、人防、食品卫生安全工作，组建应急扑救大队，完成森警部队住勤点建设，加强食品、药品安全风险监测，确保群众饮食、用药安全。严惩违法行为。坚持“四不放过”原则，加大安全生产事故的查处和责任追究力度，严肃处理责任人；切实做到“四个一律”，严厉打击非法违法生产经营建设行为。

　　切实抓好信访稳定工作。强化源头治理，加大矛盾纠纷排查密度，做到早发现、早化解，控制住、稳定好，减少信访问题的发生；加大初信初访化解力度，做到第一时间、第一地点一次性处理妥当，做到案结事了、息诉罢访；坚持多措并举，加大对进京进省非正常上访的处理力度，最大限度地减少信访积案存量。进一步创新信访工作方式方法，实行网上受理信访，健全及时就地解决群众合理诉求机制。

　　切实抓好社会治安综合治理工作。完善社会治安动态防控体系，开展严打整治行动，继续实施“法眼工程”，进一步加大设施建设投入，提升治安保障能力，不断增强公众安全感。优化中心城区交通组织，切实减轻城市交通拥堵压力。健全流动人口动态管理机制，完善特殊人群管理和服务体系。

　　（五）更加注重深化改革，增强发展内生动力

　　深化改革是加快发展的动力。一些影响发展、阻碍发展的机制性和体制性的问题都可以通过深化改革来有效解决。要在深化改革上狠下功夫，释放改革红利，激发社会活力。

　　继续深化经济体制改革。一是支持国有企业改革，做好龙煤集团鹤岗分子公司“三供一业”移交工作。二是壮大非公有制经济，制定并落实非公有制企业进入特许经营领域的办法，鼓励非公有制企业进入基础设施、金融服务、公共事业和国有企业配套协作等领域；完善和落实中小微企业发展支持政策，发挥中小微企业服务中心作用，为中小微企业提供融资、用工、法律、信息等服务。三是建立健全现代市场体系，推进工商注册制度便利化，试行“注册资本认缴制”，对部分企业有条件地允许‘零首付’出资，试行“先照后证”，允许企业“一照多址”、“一址多照”；建立城乡统一的建设用地市场，开展农村集体经营性建设用地与国有土地同等入市、同权同价试点。四是构建开放型经济新体制，推动在绥萝两县建立对俄贸易结算中心，建设面向俄罗斯及东北亚贸易投资合作商务服务基地，以建设一桥、一港、一基地为重点，扩大对俄开放。

　　继续深化农业农村改革。一是推动新型农业经营主体创新，在总结家庭农场、专业大户、农民专业合作社经验基础上，重点推进农民以土地承包经营权入股农民合作社和农业企业，发展股份合作农业和农业产业化经营。二是推动农村土地管理制度创新，加快农村土地承包经营权确权登记颁证工作，赋予农民对承包地占有、使用、收益、流转及承包经营权抵押、担保权能，鼓励承包经营权在公开市场上向专业大户、家庭农场、农民合作社、农业企业流转，发展多种形式规模经营。三是推动农村金融服务创新，鼓励金融机构延伸服务网点，广泛吸引社会资本，通过互助、合作和租赁、担保等形式，开展涉农金融服务业务。扩大土地承包经营权抵押贷款试点，推进农民住房财产权抵押、担保、转让试点。四是推动农业支持保护政策创新，深入开展涉农资金整合，加强资金监管，集中力量办大事，推动农业政策补贴向新型农业经营主体倾斜。

　　继续深化行政审批制度改革。中央和省要求取消和下放的行政审批项目，坚决取消和下放到位；没有法律法规依据和变相保留的行政审批项目，坚决取消；依据我市情况应该和可以取消的行政审批项目，坚决取消。对保留的行政审批项目要编制目录，优化流程设计，全部对社会公开。压缩企业设立和项目开工建设审批时间，提高审批效率。市政府成立企业投诉中心，及时受理各类市场主体的投诉，对影响企业发展的各类违规违纪的行政行为，要严肃查处，追究问责，公开曝光。

　　继续深化事业单位分类改革。推动公办事业单位与主管部门理顺关系和去行政化，逐步取消学校、医院等单位的行政级别。建立事业单位法人治理结构，推进有条件的事业单位转为企业或社会组织。建立事业单位统一登记管理制度。

　　按照市委、市政府《关于深化改革的意见》的要求，还要统筹推进财税体制改革、社会事业改革、文化体制机制改革、社会治理体制改革和加快生态文明制度建设。

　　（六）更加注重自身建设，打造人民满意政府

　　“打铁还需自身硬”。完成全年目标任务，必须全面加强政府自身建设，把人民满意作为最大的政治责任和政绩追求，切实做到科学理政、依法行政、高效施政和廉洁从政。

　　科学理政，进一步增强决策力。要大兴学习、调研之风，努力提高学习、研究能力。谋划工作、制定政策时要吃透上情、摸清下情、掌握实情，广泛听取吸纳社会各界的意见建议，凡是涉及经济社会发展中的重大决策，凡是与人民群众利益密切相关的重大事项，都要实行集体决策、专家咨询、公众听证和社会公示，使之更加符合客观实际、更加体现群众意愿。

　　依法行政，进一步增强公信力。政府公信力是政府的立身之本，公信力越强，就越能获得公众的信任、拥护和支持。依法行政是政府公信力的生命线，树立公信力必须从依法行政做起。要坚持依法行政，严格按照法定权限和程序行使权力、履行职责。坚持权责统一，加强权力运行的监督制约，加大行政问责力度，严肃查处失职渎职和违法行政行为，做到有权必有责、用权受监督、违法要追究。要大力推进政务公开，拓宽信息公开渠道，保障群众的知情权、参与权和表达权，自觉接受人民群众监督，始终让政府的一切权力在阳光下运行。

　　高效施政，进一步增强执行力。执行力就是政府的生命力。必须以提高执行力为突破口，全面提升政府效能。要坚决纠正消极应付、敷衍塞责、推诿扯皮的风气；坚决摒弃以会议落实会议、以文件落实文件、机械执行、照搬照抄的习惯；坚决杜绝弄虚作假、阳奉阴违、搞形式主义的现象；坚决制止变形走样、另搞一套，拒不执行、顶着不办的行为。要雷厉风行，立说立行，做到接受任务不讲条件，执行任务不找借口，对定下来的事情，要抓紧实施，及早完成；对部署了的工作，要一抓到底，确保实效；对承诺了的事项，要有诺必践，坚决兑现。

　　廉洁从政，进一步增强约束力。要始终保持艰苦奋斗、奋发进取的精神状态，严格执行《廉政准则》和廉洁从政的各项规定，加大审计监督和行政监察力度，坚决查处各类违法违纪行为。扎实推进政风行风建设，认真解决损害人民群众利益、影响发展环境的突出问题。力戒奢靡之风，反对铺张浪费，压缩政府采购、“三公”经费、会议经费、办公经费和旅差费等支出，把更多的资金用在改善民生上、用在事业发展上。

　　各位代表，鹤岗正处于加快发展的攻坚阶段，逆水行舟,不进则退。让我们在省委、省政府和市委的坚强领导下，紧紧团结和依靠全市人民，进一步解放思想、振奋精神，埋头苦干、真抓实干，为开创我市经济社会发展新局面而努力奋斗！
